# Sense of coherence, occupational stressors, and mental health among Japanese high school teachers in Nagasaki prefecture: a multiple regression analysis

**DOI:** 10.1186/s12889-020-09475-x

**Published:** 2020-09-04

**Authors:** Miho Kuwato, Yuko Hirano

**Affiliations:** 1 Nagasaki Rehabilitation College 42, Akasako, Omura, Nagasaki, 856-0048 Japan; 2 Faculty of Health Sciences, Nagasaki University 1-7-1, Sakamoto, Nagasaki, 852-8520 Japan

**Keywords:** General health questionnaire, High school teachers, Occupational stressors, Sense of coherence

## Abstract

**Background:**

International research has indicated that teachers have an increased risk of mental disorders and work-related stress, compared with those working in other fields. In Japan, the deterioration of teachers’ mental health has recently become a serious social issue. Teaching is a high-stress occupation, and job stress can affect teachers’ physical and mental health. This study aimed to determine how sense of coherence, job satisfaction, and workplace social support contribute to the mental health status of public and private high school teachers in Nagasaki Prefecture, Japan.

**Methods:**

The analytic sample comprised 370 high school teachers from eight public and three private schools in Nagasaki Prefecture who answered an anonymous survey comprising the General Health Questionnaire (GHQ-12), which is a measure of mental health status, and questions regarding sociodemographic characteristics, occupational stressors, workplace social support systems, job satisfaction, and sense of coherence. A multiple regression analysis was conducted to identify the predictors of mental health.

**Results:**

The survey was sent to 681 people, of whom 370 responded and were included in the analysis (effective response rate: 54.3%). The analysis indicated that sense of coherence was the strongest predictor of mental health (β = −.391, *p* < .0001), followed by workload stressors (β = .260, *p* < .0001), low job control (β = .099, *p* = .021), and job satisfaction (β = −.088, *p* = .040).

**Conclusion:**

The results suggest that a greater sense of coherence and job satisfaction are associated with greater mental health. Meanwhile, workload stressors and low job control undermined mental health status.

## Background

Educational environments can pose serious risks for students; these risks include bullying, truancy, and even student suicide [1]. These conditions also undermine the mental health status of teachers engaged in solving such problems. Several studies conducted in different countries internationally have reported that teachers have a higher risk of mental disorders and work-related stress, compared with other workers [2, 3].

According to a survey [4] conducted by the Japanese Ministry of Education, Culture, Sports, Science, and Technology (MEXT), teachers scored higher on fatigue and job stressors than workers from other industries (e.g., forestry, mining, construction, manufacturing, real estate and service). The main causes of stress reported were problems related to the amount of work (teachers: 60.8%; other workers: 32.3%) followed by problems with work quality (41.3, 30.4%, respectively) [4]. However, not only teaching itself but also spending long periods of time on pedagogical practices, student counseling, and miscellaneous administrative tasks associated with their work can be stressors and may undermine the mental health of teachers. Given the recent changes in Japanese educational settings, teaching has become more challenging. In the MEXT [4] summary mentioned previously, teachers were found to be facing not only issues with students who require various considerations but also with parents who demand the best for their children [4]. Due to these conditions, teachers’ overtime hours have increased, while their holidays have decreased [5].

According to a survey conducted by the Japan Trade Union Confederation, 72.9% of elementary school teachers, 86.9% of junior high school teachers, and 61.4% of high school teachers worked more than 80 h of overtime per month, which is recognized as passing the threshold of “karoshi” (literally translated as “death from overwork”) or occupational sudden mortality [6].

Teachers are exposed to various stressors under sometimes severe working conditions, resulting in deteriorating mental health status. The latest report by MEXT [7] indicated that among 920,760 teachers, sick leave was taken by 7796 public school teachers in 2018. Of these teachers, 5077 (65.1%) were diagnosed with at least one mental disorder, as formally diagnosed by a physician. Additionally, the turnover rate of public school teachers due to mental health disorders has remained high: 57.6% in elementary schools, 58.1% in junior high schools, and 58.8% in senior high schools [4]. In private schools, the turnover rate was 47.4% for elementary schools, 42.4% for junior high schools, and 37.9% for senior high schools [4].

Various factors cause the stress experienced by teachers, and poor physical and mental health can affect teachers’ ability to perform their duties [5]. Further, if teachers continue working while having impaired mental health or develop a mental illness requiring a leave of absence, the management of the school may be affected [5]. Thus, developing methods to maintain and improve high school teachers’ mental health is urgently needed. Occupational stressors are one factor having a negative impact on the mental health of teachers. According to existing research, some of the stressors that affect the mental health of teachers are job content stress [8], long working hours [9], job dissatisfaction [10–12], interpersonal conflict at work [13, 14], conflict with students and parents [13], and high job demands with low job management [15, 16]. However, most of these existing studies have focused on primary school and junior high school teachers, with few focusing on high school teachers [9, 11, 12, 14–16]. Moreover, the educational and career guidance needed by high school students is more specialized than that required for elementary and junior high school students [17]. Thus, differences in the stress structure between high school teachers and those working in elementary and junior high schools might exist. Previous research that investigated teacher stress by school type indicated that high school teachers had higher stress levels and received less social support than elementary school and junior high school teachers [18]. Therefore, this study aimed to examine private and public high school teachers.

Despite these sometimes severe working conditions, many teachers are able to manage their stress and even perform well, carrying out their duties without experiencing deterioration of their mental health. Another aspect affecting teachers’ mental health involves the factors that enable teachers to maintain positive mental wellbeing. We hypothesized that sense of coherence (SOC) [19], job satisfaction, and social support might positively affect teachers’ mental health status.

SOC is a key concept of stress-coping behavior. It comprises three components: “comprehensibility,” “manageability,” and “meaningfulness.” [19] As individuals cope with stress, SOC mobilizes psychosocial resources known as generalized resistance resources [19]. Previous studies have suggested that higher SOC levels may protect mental health, regardless of location, occupation, age group, and gender [20–25]. Additionally, job satisfaction contributes to mental health, regardless of occupation type [10–12, 26, 27]. Moreover, social support creates conditions that help individuals cope with stressful situations [28]. This is especially true among teachers, as social support has been found to help maintain mental health and avoid burnout [29], while also acting as a buffer for depression [15]. Additionally, job satisfaction contributes to mental health regardless of location (e.g., Iran, Japan, South East Nigeria, Thailand) [10–12] or occupation type (e.g., factory worker, school teachers, local civil servants) [10–12, 26, 27].

In this study, we examined how SOC, job satisfaction, and workplace social support contribute to mental health status when mental health status is undermined by occupational stressors. We considered four types of occupational stressors: workload stress generated by the work itself; high job demands due to time constraints and quantitative load; low participation in job decision-making, including low job control; and interpersonal environment stressors generated from the workplace atmosphere and interpersonal relationships.

## Methods

### Participants and procedures

The target population of this study comprised all public and private high school teachers in Nagasaki Prefecture, which included 2178 teachers from 54 public high schools and 820 teachers from 22 private high schools. Teachers who were on sick leave, maternity leave, and childcare leave during the survey period were excluded.

All of the full-time high schools in Nagasaki Prefecture were divided into three school districts (south, north, and central). Next, they were divided according to size into small schools (11 classes or fewer), medium-sized schools (12–18 classes), and large schools (19 classes or more). Subsequently, we randomly selected one public school and one private school of each size from the three school districts and invited a total of 18 schools to participate in the survey. Ultimately, we surveyed eight public schools and three private schools from those selected that agreed to cooperate. After obtaining written consent from the principal of each high school, an anonymous questionnaire was distributed to each high school teacher (*n* = 681). The survey was conducted from December 1 to 18, 2017.

### Sample size determination

We used a single population proportion equation [30] to calculate the appropriate sample size required for this study. We estimated the minimum sample size (*n*) required according to the following parameters: the response rate (p) was set at .5, which required the maximum number of surveyed persons, the sample error (d) was 5%, and the confidence interval was 95% (λ = 1.96).

$$ n=\left( z\alpha /2\right)/2p\ \left(1-p\right)\ d2=(1.96)\ 2\times .5\ \left(1-.5\right)\ (.05)\ 2=384.16 $$

Thus, the minimum sample size was calculated as 384 respondents. To account for the anticipated non-response rate, the questionnaire was sent to 681 teachers.

### Ethical considerations

The study was conducted with the approval of the Ethics Review Committee of the Graduate School of Biomedical Sciences, Nagasaki University (No. 17101298). Participation in the study was voluntary, and all participants provided written informed consent. All information was collected anonymously, and the data were used for research purposes only.

### Measures

The sociodemographic data gathered via questionnaire comprised gender, age, employment status (permanent or part-time), school type (public or private), number of hours worked per week, and whether teachers currently engaged in supervising club activities, held a managerial position, and were homeroom teachers. In the analysis, these were selected as control variables.

Perceived occupational stressors included four groups: workload stressors, interpersonal stressors, high job demands, and low job control.

The Workload Stressor Scale, comprising 13 items, was created by modifying the Simplified Mental Health Questionnaire for Teachers developed by MEXT [4]. The scale included questions about the burden of pedagogical practice, student counseling, supervising club activities, administrative work, and student recruitment activities. It also asked about stressors related to relationships with co-workers, upper management, and parents, as well as stressors related to school policies, salary and compensation, and personal lives. The degree of excess quality and quantity of duties was also included in the scale. Items were rated on a 4-point Likert scale from 1 (*few stressors*) to 4 (*many stressors*). A total score was generated after adding all items on the scale (score range: 13–52). Higher scores indicated a higher number of reported stressors. The Cronbach’s α was .879.

The Interpersonal Stressor Scale comprised nine items and was created by modifying the Simplified Mental Health Questionnaire for Teachers developed by MEXT [4]. It included questions such as “Is the atmosphere of the office cheerful?” “Are the teachers at your workplace cooperative?” and “Are there activities to improve the working environment?” Items were rated on a 4-point Likert scale from 1 (*strongly disagree*) to 4 (*strongly agree*). A total score was calculated by adding all items on the scale (score range: 9–36). Higher scores indicated higher levels of stress experienced. The Cronbach’s α was .841.

High job demands were measured using 12 items of the Brief Job Stress Questionnaire [31], including “I must deal with a lot of work” and “I cannot finish my work within my regular working hours.” Items were rated on a 4-point Likert scale from 1 (*strongly disagree*) to 4 (*strongly agree*). The tool has been translated into Japanese and validated [31]. A total score was calculated by adding all items on the scale (score range: 2–8). Higher scores indicated more extreme job demands. The Cronbach’s α was .702.

Degree of job control was measured using 12 items of the Brief Job Stress Questionnaire [31], including “I can work at my own pace,” “I can decide the sequence and method of my work by myself,” and “My opinion is reflected in the workplace policy.” The items were rated on a 4-point Likert scale from 1 (*strongly agree*) to 4 (*strongly disagree*). A total score was calculated by adding all items on the scale (score range: 3–12). Higher scores indicated lower job control. The Cronbach’s α was .726.

The Brief Scale of Social Support for Workers (short version) [32] measures social support from colleagues and supervisors in the workplace. The Japanese translation of this tool has been validated [32]. It contains six questions: “Do you have a person that you can ask for a consultation?” “Do you have anyone to ask for advice on solving problems?” “Do you have someone to follow when you do your job?” “Do you have someone who supports your actions and ideas?” “Do you have someone to deal with together?” and “Do you have someone who understands and appreciates you?” The questions are answered on a 4-point Likert scale from 1 (*not at all*) to 4 (*always*). A total score was calculated by adding all items on the scale (score range: 6–24). The higher the score, the more social support was reported. The reliability and validity of the Japanese translation of the scale have been previously verified [32], and the Cronbach’s α was .942.

The degree of job satisfaction was measured with the single question, “Are you satisfied with your current job?” answered on a 4-point Likert scale from 1 (*not satisfied at all*) to 4 (*very satisfied*). A higher score indicated greater satisfaction with the current job.

The Japanese version of the 13-item Sense of Coherence Scale (SOC-13) was used to measure the ability to cope with stress. The scale’s 13 items are rated on a 7-point Likert scale, with higher scores indicating a stronger SOC. A total score was calculated by adding all items on the scale (score range: 13–92). Previous studies verified the reliability and validity of the scale [33]. The Cronbach’s α was .839.

We used the Japanese version of The General Health Questionnaire (GHQ-12) [34] to measure respondents’ mental health status. The scale is standardized to evaluate mental health status. In this study, we applied 0–1–2-3 coding to assess the degree of mental health. The total score was calculated by adding all items on the scale (range: 0–36). A higher score indicated poorer reported mental health. The Cronbach’s α was .895.

### Statistical a1nalysis

We used SPSS v 25 for statistical analyses. Descriptive statistics, one-way analysis of variance (ANOVA), and independent-sample *t*-test were used to compare the mean GHQ-12 scores according to demographic characteristics. Pearson’s correlation coefficient was used to test the associations between variables and the GHQ-2 score. Multiple regression analysis was employed to determine the most appropriate model for predicting the GHQ-12 score. We used a variance inflation factor (VIF) to confirm that multicollinearity did not occur between the explanatory variables (1.21 < VIF < 2.04). The level of statistical significance was set at *p* < .05.

## Results

The survey was sent to 681 people, of whom 370 responded and were included in the analysis (effective response rate: 54.3%). To determine the representativeness of our sample, we compared its age and gender distributions to those of the entire population of Japanese high school teachers. The number of Japanese high school teachers was 211,857 males, accounting for 68.7% of the population, and the average age was 45.3 [35]. In this study, men accounted for 71.6% of participants and the average age was 46.3.

The sociodemographic characteristics of the respondents and the relationship between the sociodemographic variables and the GHQ-12 score are shown in Table 1. A significant difference was observed between age groups, *F* (4,365) = 2.489, *p* = .043; participants in their 20s showed the highest GHQ-12 scores (15.3 ± 4.8), while those in their 60s showed the lowest (12.2 ± 4.0). Regarding employment status, permanent employees had significantly higher GHQ-12 scores (14.1 ± 5.5), compared with part-time employees (12.6 ± 3.8). Regarding working hours, the GHQ-12 score was significantly correlated with the number of hours worked per week (*r* = .174, *p* = .001).

**Table 1:** Relationship between baseline characteristics and General Health Questionnaire-12 (GHQ-12) scores (*N* = 370)

					GHQ-12 scores		
			*N*	%	Mean	SD	*p*-value
Gender	Male		265	71.6	13.70	5.3	.294^a^
	Female		105	28.4	14.30	5.4	
Age (years)	Mean ± SD		46.3 ± 10.4				
	20s		30	8.1	15.30	4.8	.043^b^
	30s		59	15.9	12.70	5.4	
	40s		128	34.6	14.20	5.0	
	50s		118	31.9	14.30	5.9	
	60s		35	9.5	12.20	4.0	
Employment status	Part-time		59	15.9	12.60	3.8	.042^a^
	Permanent		311	84.1	14.10	5.5	
School type	Public		288	77.8	13.80	5.2	.674^a^
	Private		82	22.2	14.10	5.6	
Manager	No		345	93.2	13.80	5.4	.177^a^
	Yes		25	6.8	15.00	4.3	
Engaged as club advisor	No		67	18.1	13.80	5.4	.555^a^
	Yes		303	81.9	14.20	4.9	
Engaged as homeroom teacher	No		196	53.0	14.30	5.7	.216^a^
	Yes		174	47.0	13.60	5.0	
Weekday working hours (minutes)	Mean ± SD		705.4 ± 77.0				
Job social support	Mean ± SD		18.7 ± 3.6				
SOC-13 score	Mean ± SD		60.1 ± 11.5				
GHQ-12 score	Mean ± SD		13.9 ± 5.3				

*SOC-13* 13-item Sense of Coherence scale

^a^*t*-test ^b^; one-way ANOVA

Table 2 shows the associations between each variable and the GHQ-12 scores. Significant positive correlations were found between the GHQ-12 and workload stressors, interpersonal stressors, high job demands, and low job control.

**Table 2:** Correlation and multiple regression analysis of various factors in relation to General Health Questionnaire-12 (GHQ-12) scores (*N* = 370)

	Pearson correlation		Standard partial regression coefficient	
	*r*	*p*	β	*P*
Gender (M: 0, F: 1)	.055	.294	.009	.819
Age (20s: 1, 30s: 2, 40s: 3, 50s: 4, 60s: 5)	−.038	.461	.068	.095
Employment status (Part-time: 0, Permanent: 1)	.106	.042	.028	.473
School type (Public: 0, Private: 1)	.023	.660	−.047	.241
Manager (No: 0, Yes: 1)	.059	.260	−.006	.887
Engaged as club advisor (No: 0, Yes: 1)	−.029	.578	−.034	.464
Engaged as homeroom teacher (No: 0, Yes: 1)	.065	.213	−.025	.529
Weekday working hours (Minutes)	.174	.001	.045	.302
Workload stressors	.603	<.0001	.260	<.0001
Interpersonal environment stressors	.489	<.0001	.055	.278
High job demands	.414	<.0001	.067	.154
Low job control	.423	<.0001	.099	.021
Job satisfaction	−.422	<.0001	−.088	.040
Job social support	−.377	<.0001	−.021	.654
SOC-13 scores	−.663	<.0001	−.391	<.000
*R*^2^			.564	<.0001
Adjusted *R*^2^			.545	

Negative correlations were found between the GHQ-12 scores and SOC, job satisfaction, and social support in the workplace. Multiple regression analysis indicated that SOC was the strongest predictor of the GHQ-12 score, followed by job stressors, low job control, and work satisfaction.

## Discussion

The purpose of this study was to determine which factors had an effect on the mental health of high school teachers. The results indicated that the strongest predictor of mental health was SOC, followed by workplace stressors, low job control, and job satisfaction.

Our results indicated that SOC contributes to maintaining the positive mental health of high school teachers. This finding is consistent with previous studies that examined various populations and professions, including nurses in Lithuania [20], Japanese male nurses,[21] information technology company workers [22], manufacturers [23], software engineers in Japan [24], and municipal government officers about to retire [25]. The results from these studies indicate that SOC may affect mental health status regardless of age [25], gender [21], country [20, 21], or occupation [20, 22–25]. SOC appears to have a greater effect on mental health in situations where there are many stressors, such as working environment stress, workload-related stressors, and low job control [19]. Therefore, it is worth improving the external environment—including workload stressors—as well as strengthening the internal, individual factors of SOC.

Further, job satisfaction was inversely associated with a lower GHQ-12 score, suggesting that higher job satisfaction was related to fewer self-reported mental health challenges. This has also been demonstrated by previous studies among populations such as factory workers in the south of Thailand [26], teachers in Marand, Iran [10], school teachers in Japan [11], secondary school teachers in southeast Nigeria [12], and local civil servants in Japan [27].

Among the 920,760 teachers in Japan, 7796 public school teachers took sick leave in 2018, of which 5077 (65.1%) were diagnosed with mental health disorders [7]. The present study confirms that workload stressors negatively affect the mental health of high school teachers in Japan [8, 14, 36–38], and this is true in other countries as well [39–41].

According to a survey conducted by the Japan Trade Union Confederation, 72.9% of elementary school teachers, 86.9% of junior high school teachers, and 61.4% of high school teachers worked more than 80 h of overtime per month, which is recognized as exceeding the accepted threshold for karoshi (occupational sudden mortality) [6]. Teachers report that long working hours have a negative impact on their mental health [9, 42, 43]. In addition, long working hours may be among the causes behind the 109 cases of karoshi among teachers between 2010 and 2016, according to the Ministry of Internal Affairs and Communications (2019) [44]. This should alert officials that Japanese teachers are being exposed to working conditions that undermine their mental health. The Ministry statistics do not include cases of karoshi among private high school teachers; therefore, the total number of such cases is likely to be even higher. As an emergency measure, MEXT launched “The Work Style Reform in Schools” to manage teachers’ working hours, including outsourcing some club activities to local communities [45]. These suggestions may decrease working hours and increase teachers’ job control by freeing them from miscellaneous tasks; as a result, they may be able to concentrate on teaching at their own pace, which will enable them to better maintain their mental health.

### Limitations of this study

This study was conducted in Nagasaki Prefecture, a remote area of Japan; therefore, the results may not generalize to other populations of teachers or those in other countries. Furthermore, as participation in this survey was voluntary, the relatively low response rate of busy teachers might have contributed to sample bias. Additionally, the current sample was slightly older and more likely to be male than the overall population of Japanese high school teachers, which may have biased the results. Further research using larger samples is therefore needed. Additionally, extending the target population beyond Japan will improve the generalizability of the results.

## Conclusion

This study identified the factors predicting mental health among high school teachers. The results suggest that higher SOC and job satisfaction are associated with better mental health, while workload stressors and low job control undermine mental health. Actively promoting initiatives to reduce job stressors and improve the working environment is critical for maintaining and improving the mental health of high school teachers. Further, it is necessary to provide teachers with support to improve their SOC and job satisfaction. The number of teachers should be increased, the workload should be reduced, and support staff should be assigned to promote the mental health of high school teachers and allow them to focus on the tasks at hand. The mental health of teachers should be recognized as being both a physical and mental health problem for individual teachers and a health problem for the school as a whole and the community. Thus, promoting the creation of a work environment that considers the mental health of teachers is extremely important. Moreover, teachers need to recognize the importance of their mental health and implement preventive mental health measures. One potential strategy is the development of a stress management program that addresses the three elements of SOC (i.e., “comprehensibility,” “manageability,” and “meaningfulness”), an important concept of stress-coping behavior. We considered this to be useful to ensure teachers’ mental health. Further research is needed to promote mental health measures for teachers based on the current state of various schools.

## Data Availability

Participants shared their opinions and experiences upon the assurance that their confidentiality and anonymity would be protected. Hence, the research data is not available publicly, as this would compromise individual privacy and our ethical approval conditions. However, the datasets used and analyzed during the current study are available from the corresponding author on reasonable request.

## Abbreviations

GHQ: General Health Questionnaire
SOC: Sense of coherence
MEXT: Japan’s Ministry of Education, Culture, Sports, Science, and Technology
